# Benchmarking of Data-Driven Causality Discovery Approaches in the Interactions of Arctic Sea Ice and Atmosphere

**DOI:** 10.3389/fdata.2021.642182

**Published:** 2021-08-24

**Authors:** Yiyi Huang, Matthäus Kleindessner, Alexey Munishkin, Debvrat Varshney, Pei Guo, Jianwu Wang

**Affiliations:** ^1^ Department of Hydrology and Atmospheric Sciences, University of Arizona, Tucson, AZ, United States; ^2^ Paul G. Allen School of Computer Science and Engineering, University of Washington, Seattle, WA, United States; ^3^ Department of Computer Science and Engineering, University of California Santa Cruz, Santa Cruz, CA, United States; ^4^ Department of Information Systems, University of Maryland, Baltimore, MD, United States

**Keywords:** causality discovery, time series, arctic sea ice, temporal causality discovery framework, non-combinatorial optimization *via* trace exponential and augmented lagrangian for structure learning, directed acyclic graph-graph neural networks, atmosphere-sea ice interactions

## Abstract

The Arctic sea ice has retreated rapidly in the past few decades, which is believed to be driven by various dynamic and thermodynamic processes in the atmosphere. The newly open water resulted from sea ice decline in turn exerts large influence on the atmosphere. Therefore, this study aims to investigate the causality between multiple atmospheric processes and sea ice variations using three distinct data-driven causality approaches that have been proposed recently: Temporal Causality Discovery Framework Non-combinatorial Optimization *via* Trace Exponential and Augmented lagrangian for Structure learning (NOTEARS) and Directed Acyclic Graph-Graph Neural Networks (DAG-GNN). We apply these three algorithms to 39 years of historical time-series data sets, which include 11 atmospheric variables from ERA-5 reanalysis product and passive microwave satellite retrieved sea ice extent. By comparing the causality graph results of these approaches with what we summarized from the literature, it shows that the static graphs produced by NOTEARS and DAG-GNN are relatively reasonable. The results from NOTEARS indicate that relative humidity and precipitation dominate sea ice changes among all variables, while the results from DAG-GNN suggest that the horizontal and meridional wind are more important for driving sea ice variations. However, both approaches produce some unrealistic cause-effect relationships. Additionally, these three methods cannot well detect the delayed impact of one variable on another in the Arctic. It also turns out that the results are rather sensitive to the choice of hyperparameters of the three methods. As a pioneer study, this work paves the way to disentangle the complex causal relationships in the Earth system, by taking the advantage of cutting-edge Artificial Intelligence technologies.

## 1 Introduction

Warming in the Arctic has been much faster than in the rest of the world in both observations and model simulations, a phenomenon known as the Arctic amplification (Holland and Bitz, 2003; Serreze and Barry, 2011). Decline in sea ice has occurred in all seasons, which is believed to be the major driver of Arctic amplification. Over the last few decades, Arctic summer sea ice extent has declined by nearly 50% with accelerated retreat in the early 21st century (Serreze and Stroeve, 2015; Simmonds, 2015). These dramatic changes in the Arctic sea ice affect a growing community of diverse stakeholders. Accompanying this growing interest is an urgent demand to increase the pace and scope of the advancements in physical understanding and predictive capabilities. As one of the most important components in the Earth System, the atmosphere actively interacts with the sea ice underneath. On the one hand, the sea ice variations are caused by different dynamic and thermodynamic forcings. On the other hand, sea ice decline in turn exerts large influence on the atmosphere. This will further alter the climate patterns in both Arctic and mid-latitudes, which results in more frequent extreme weather events (Cohen et al., 2014; Simmonds and Govekar, 2014; Sun et al., 2016; Yao et al., 2017; Luo et al., 2018; Luo et al., 2019a; Luo et al., 2019b). These two-way feedbacks are potentially very important in terms of understanding the Arctic warming in the past and future. In most cases, these connections are highly nonlinear and conditionally constrained (e.g., differ by season or region), making them even more complex. For example, a link between recent winter sea ice decline and mid-latitude cold extremes could be mediated by whether there is a weak gradient of background potential vorticity (Luo et al., 2019b; Luo et al., 2018; Luo et al., 2019a). Therefore, it is vital to analyze both the sea ice retreat’s influence on the atmosphere and vice versa.

The traditional way to discover causal relations is to manipulate the value of a variable by using interventions or real-life experiments. All other influencing factors of the target variable can be held fixed, to test whether a manipulation of a potential cause changes the target variable (Nauta et al., 2019). Specifically, the typical approach for assessing causal links in climate study is targeted modeling experiments. Such experiments are often computationally expensive, time-consuming, or even impossible to carry out. More importantly, the large biases and substantial model spread remain in the state-of-the-art climate models (Stocker et al., 2013), which further introduce some unrealistic causal relations. With the current advances in digital sensing and data assimilation, we have entered a period where Earth science tends to be data rich in observations (Overpeck et al., 2011), allowing us to do data-driven causality discovery (Guo et al., 2020; Zhang et al., 2018; Nauta et al., 2019). The data-driven causality approach aids scientists in identifying and extracting signals by analyzing statistical properties of purely observational data, which augments targeted model studies and has direct ties to forecasting and prediction. For time-series data, many popular data-driven causality frameworks are proposed such as Granger Causality (Granger, 1969), PC Momentary Conditional Independence (PCMCI) (Runge et al., 2019), Time Series Models with Independent Noise (TiMINo) (Peters et al., 2012), Additive Non-linear Time Series Model (ANLTSM) (Chu and Glymour, 2008) and time series Fast Causal Inference (tsFCI) (Entner and Hoyer, 2010). Several different frameworks for observational analysis have been applied to climate science to provide graphical representations of causal relations. For example, Ebert-Uphoff and Deng (2012) investigated causal relationships between four prominent modes of atmospheric low-frequency variability in boreal winter using Graphic Models. McGraw and Barnes (2018) highlighted the Granger Causality by a simple Monte Carlo example. More recently, Song et al. (2018), Song et al. (2019) demonstrated the Granger causality between El Niño and the southern oscillation (ENSO) and other climate variables. Some other applications in climate sciences include Chu et al. (2005); Zerenner et al. (2014); Hussung et al. (2019). Among them, the most relevant topic is the connections between Arctic and mid-latitude climate patterns, such as the Arctic drivers of mid-latitude winter circulations (Kretschmer et al., 2016; Samarasinghe et al., 2019) as well as the impacts of Arctic sea ice on circulations in the North Atlantic Ocean (Strong et al., 2009) and Western Pacific (Matthewman and Magnusdottir, 2011). However, neither study investigates the relationship between sea ice retreat and the atmospheric dynamic and thermodynamic processes in the Arctic only, which is the focus of this study. It is unclear whether different causality approaches would produce similar results, or whether a particular technique is best suited for this topic as each study employs a different approach. Moreover, it is also valuable to evaluate whether these data-driven causality discovery approaches could capture those conditional and threshold-related connections.

Thus, the overarching goal of this study is to investigate the causality between multiple atmospheric processes and sea ice variations from sub-seasonal to seasonal timescales using data-driven causality approaches. Instead of performing multiple climate model simulations, here we focus solely on an observational-type analysis. Specifically, three distinct data-driven causality approaches, Temporal Causality Discovery Framework (TCDF) (Nauta et al., 2019), Non-combinatorial Optimization via Trace Exponential and Augmented lagrangian for Structure learning (NOTEARS) (Zheng et al., 2018) and Directed Acyclic Graph-Graph Neural Networks (DAG-GNN) (Yu et al., 2019), will be used and compared to determine whether they are suitable for the particular climate study. The main reasons we chose these three approaches are: 1) the three approaches are relatively new (published in 2018 or later) and we have not seen studies applying or evaluating them with climate data; 2) both TCDF and DAG-GNN are deep learning based approaches and deep learning approaches normally can learn nonlinearity from datasets better than traditional machine learning approaches (Schmidhuber, 2015). Because DAG-GNN is built on top of NOTEARS for nonlinearity mapping, we included NOTEARS to see whether DAG-GNN can do better than NOTEARS for our dataset.

This paper is structured in the following sections. Section 2 summarizes the main conclusions from previous studies in terms of causal relations between different atmospheric processes and Arctic sea ice variations; Section 3 lists data sets and data pre-processing methods and steps; Section 4 introduces three data-driven causality discovery frameworks; Section 5 summarizes the results generated by each method and compares those results with a causality graph based on literature review. Finally, Section 6 reports the main conclusions and limitations of this study.

## 2 Causality Between Atmospheric Processes and Arctic Sea Ice Variations

**FIGURE 1:**
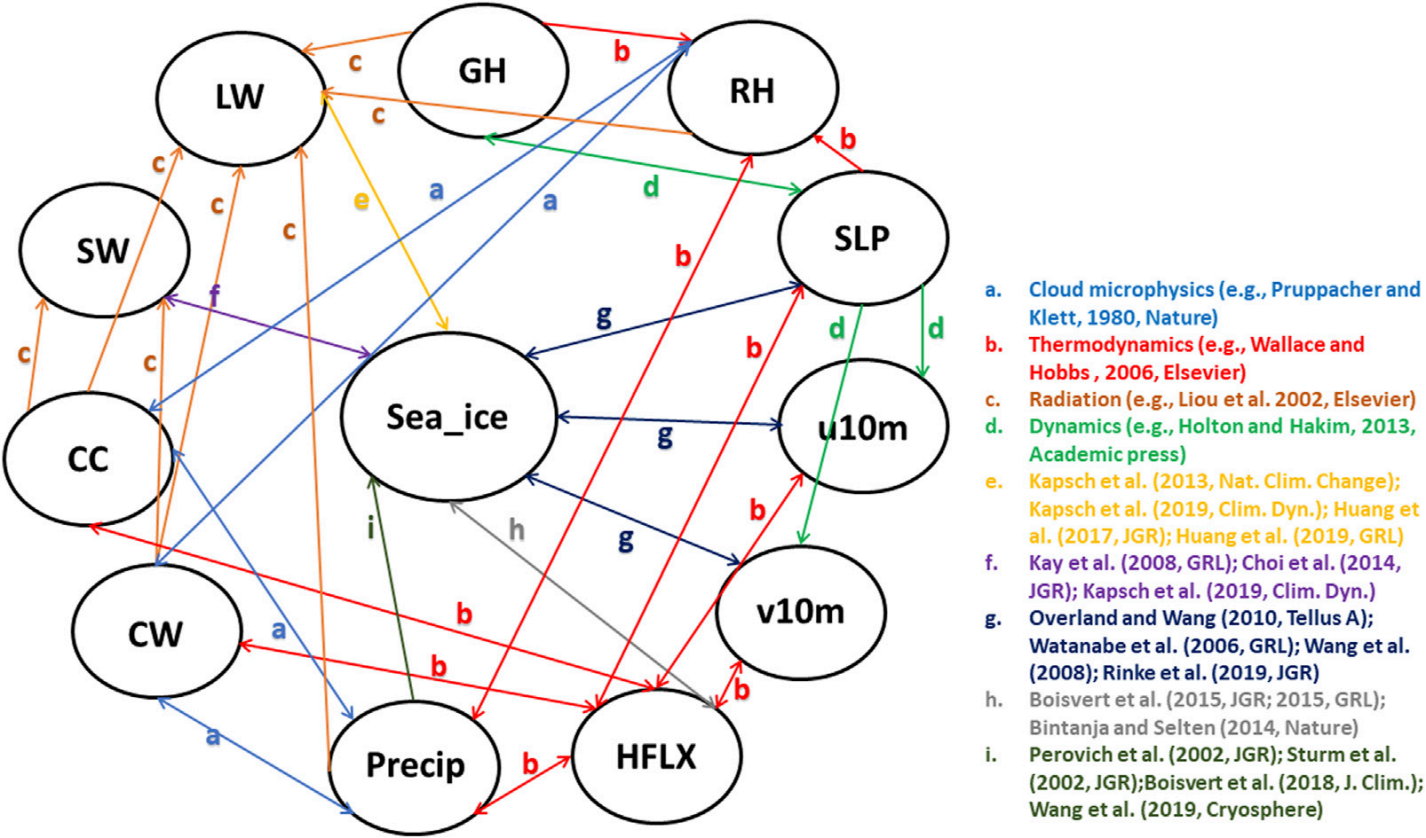
The causality graph between key atmospheric variables and sea ice over the Arctic based on literature review. This graph represents the domain knowledge. Note that the processes *a* − *d* are well-known atmospheric processes, which can be summarized from multiple textbooks. The processes *e* − *i* are summarized from recent peer-reviewed publications and they are ongoing research. The *sea*_*ice* here represents sea ice coverage and/or sea ice thickness; *GH* is the geopotential height; *RH* is relative humidity; *SLP* means sea level pressure; *u*10*m* and *v*10*m* represents meridional and zonal wind at 10 m, respectively; *HFLX* is the sensible plus latent heat flux; *Precip* is the total precipitation; *CW* is the total cloud water path; *CC* is the total cloud cover; *SW* and *LW* represent net shortwave and longwave flux at the surface, respectively.

Due to the two-way interactions between the atmosphere and sea ice, studying causality between them is a challenging but important task, which makes it an area of high interest within polar climate community. The sea ice variations can be caused by different dynamical and thermodynamical processes. Important dynamical processes include anomalous surface wind (Spreen et al., 2011; Wu et al., 2012), regional atmospheric circulation patterns (Overland and Wang, 2010; Screen et al., 2018; Rinke et al., 2019) and abnormal storm activities (Simmonds et al., 2008; Simmonds and Keay, 2009; Screen et al., 2011; Simmonds and Rudeva, 2012; Parkinson and Comiso, 2013; Simmonds and Rudeva, 2014). Cloud (Kapsch et al., 2013), radiation (Kay and Gettelman, 2009; Choi et al., 2014) and precipitation (Boisvert et al., 2018; Wang et al., 2019; Marcovecchio et al., 2021) are the important thermodynamic factors controlling Arctic sea ice trends and variability. On the other hand, sea ice decline in turn exerts large influence on the atmosphere, including cloud (Kay and Gettelman, 2009; Morrison et al., 2018), surface energy budget (Semmler et al., 2012; Boisvert et al., 2015; Boisvert and Stroeve, 2015), precipitation (Bintanja and Selten, 2014; Kopec et al., 2016) and large-scale circulation (Chemke et al., 2019; Kennel and Yulaeva, 2020). Figure 1 depicts the causal relations between key atmospheric variables and sea ice over the Arctic. The sea ice here represents sea ice coverage and/or sea ice thickness. Note that the processes *a* − *d* are well-known atmospheric processes, including cloud microphysics, thermodynamics, radiation, climate dynamics, which have been studied over the past few decades. The processes *e* − *i* are summarized from more recent publications, which are still under investigation by climate scientists. We will explain processes *e* − *i* in details in the next paragraph.

The arrow *e* represents the two-way effect between sea ice and net longwave flux at the surface. Based on global reanalysis (Kapsch et al., 2013; Lee et al., 2017), surface (Cox et al., 2016) and satellite observations (Huang et al., 2017), as well as model simulations (Kapsch et al., 2016; Huang et al., 2019a), the downward longwave radiation at the surface dominates surface warming and therefore enhances sea ice melt in winter and spring. The increase in downward longwave flux is a result of an increase in cloudiness and moisture in the Arctic Basin, which is caused by enhanced local evaporation or moisture transport from mid-latitudes (Luo et al., 2017). Positive anomalies of longwave flux in spring and early summer initiate an earlier melt onset, thereby triggering several feedback mechanisms which amplify melt during the succeeding months (Kapsch et al., 2016; Huang et al., 2019b). The sea ice melt increases the air temperature and thus increases the longwave flux at the surface. The downward shortwave flux, however, appears only important after the melt has started (Kapsch et al., 2013; Huang et al., 2017). Once the surface albedo is significantly reduced due to sea ice melt, the solar radiation could be absorbed by ocean, which further accelerates ice melt in late spring and summer (Kay and Gettelman, 2009; Choi et al., 2014; Kapsch et al., 2016). The feedback between surface net shortwave flux and sea ice, represented by arrow *f* in Figure 1, has been confirmed by both model simulations (Kapsch et al., 2016) and satellite observations (Kay and Gettelman, 2009; Nussbaumer and Pinker, 2012; Choi et al., 2014). The arrows *g* indicate the interactions between the sea ice variations and atmospheric dynamical processes. A series of studies demonstrated that recent loss of Arctic sea ice is triggered by the atmospheric circulation changes such as a tendency toward a dipole pattern in the mean sea level pressure trend with an increase over the Arctic Ocean and a decrease over Siberia. The Arctic dipole anomaly in summer (Wang et al., 2009), winter (Watanabe et al., 2006) and spring (Kapsch et al., 2019) produces a strong meridional wind (v-component) anomaly that drives more sea ice out of the Arctic Ocean. In addition, this dipole anomaly promotes transport of heat and moisture and thus enhances downward longwave radiation and control the melt onset (Huang et al., 2019b; Kapsch et al., 2019). Moreover, the changes in cyclone occurrence and/or depth during spring (Screen et al., 2011) and summer (Simmonds and Rudeva, 2012) have preconditioning effects on the sea ice cover and exert a strong influence on the amount of sea ice that survives the melt season. A recent study also pointed out that a stronger anticyclonic circulation over Greenland and the Arctic Ocean in the troposphere may have contributed as much as 60% to the September sea ice extent decline since 1979, by warming and moistening the lower atmosphere (Ding et al., 2017; Huang et al., 2021; Luo et al., 2021). On the other hand, the reduction in Arctic sea ice extent and increase in open water area in late summer are found to directly contribute to a modification of large-scale circulation patterns in the following autumn through the additional heat stored in the Arctic Ocean and released to the atmosphere during the autumn (Overland and Wang, 2010). The increased 1,000–500 hPa thickness in autumn produce anomalous easterley zonal wind component (u-component), especially over the north of Alaska and Canada. Moreover, a more meridional flow pattern associated with sea ice reduction have an impact on the mid-latitude weather (Overland and Wang, 2010). These conclusions are mainly drawn from model simulations (Watanabe et al., 2006; Rinke et al., 2019), reanalysis and observations (Wang et al., 2009; Overland and Wang, 2010; Kapsch et al., 2019). In addition to radiation, the sensible plus latent heat flux also plays an important role in the Arctic energy budget. The increase in the downward moisture flux triggers the melting of the sea ice in spring (Boisvert et al., 2015; Boisvert and Stroeve, 2015; Marcovecchio et al., 2021). Earlier melt onset and loss of sea ice in the spring enhance warming of the ice-free ocean surface, which in turn leads to an increase of evaporation from the surface into the atmosphere in the autumn. This positive feedback between heat flux and sea ice, indicated by arrow *h*, has been confirmed by satellite observations (Boisvert et al., 2015; Boisvert and Stroeve, 2015) and model simulations (Huang et al., 2019a) during most months of the year. The arrow *i* represents the influence of precipitation on Arctic sea ice variations. Specifically, the magnitude of precipitation accumulating over the sea ice pack largely determines the depth of the snow layer, which modulates the rate of sea ice growth because of its highly insulating properties (Sturm et al., 2002). The phase of the precipitation falling on the sea ice pack is also important. As rain, it can melt, compact, and densify the snowpack, thus reducing the surface albedo and promoting sea ice melt (Perovich et al., 2002).The recent snowfall decline in summer is essentially caused by changes in precipitation form (snow turning to rain) with very little influence of decreases in total precipitation, which is a result of lower-atmospheric warming. Then the loss of snow-on-ice results in a substantial decrease in the surface albedo over the Arctic Ocean, causing additional surface ice melt by absorbing more solar radiation (Screen and Simmonds, 2012). These conclusions are mainly drawn from *in-situ* measurements during field campaign (Perovich et al., 2002; Sturm et al., 2002), global reanalysis products and surface observations (Screen and Simmonds, 2012; Boisvert et al., 2018; Wang et al., 2019). The higher precipitation and snowfall could result in a thicker snowpack that allows less heat loss to the atmosphere. More importantly, modeling studies suggest that increases in Arctic precipitation over the 21st century, particularly in late autumn and winter, are due mainly to strongly intensified local surface evaporation (latent heat flux) (Bintanja and Selten, 2014). Therefore, we believe that Arctic precipitation exerts direct influence on sea ice variations (arrow *i*), while sea ice modulates precipitation mainly through sensible plus latent heat flux (arrows *h*, *b*).

Among these studies, very few of them have demonstrated the delayed impact of one variable on another. Specifically, the net shortwave flux at the surface in early summer (May–July) is found to enhance sea ice melt with a lag of 1–4 months (Choi et al., 2014). Moreover, the sea ice condition exhibits the delayed impacts on itself, which is called sea ice anomaly persistence (Guemas et al., 2016; Cruz-García et al., 2019; Holland et al., 2019). The sea ice anomaly persistence depends on the predictand (area, extent, volume), region, and the initial and target dates, which can be varied from a few days to a few years (Guemas et al., 2016). With sea ice anomaly persistence, there is predictability for the sea ice area in winter but low predictability throughout the rest of the year in peripheral seas. Based on multiple model simulations, the Labrador Sea stands out among the considered regions, with sea ice predictability extending up to 1.5 years (Cruz-García et al., 2019).

Note that most of studies mentioned above determine the changes in one variable, happening before another one, by applying time series analysis and/or composite analysis based on observations, reanalysis or model output (Perovich et al., 2002; Sturm et al., 2002; Kay and Gettelman, 2009; Wang et al., 2009; Nussbaumer and Pinker, 2012; Kapsch et al., 2013; Choi et al., 2014; Boisvert et al., 2015; Boisvert and Stroeve, 2015; Cox et al., 2016; Huang et al., 2017; Huang et al., 2019b; Boisvert et al., 2018; Wang et al., 2019). Among them, some studies use more advanced statistical analysis such as empirical orthogonal function (Watanabe et al., 2006; Overland and Wang, 2010) and self-organizing map (Kapsch et al., 2019; Rinke et al., 2019). Other studies assess the causal links through targeted modeling experiments (Bintanja and Selten, 2014; Kapsch et al., 2016; Ding et al., 2017; Huang et al., 2019a; Cruz-García et al., 2019), in order to test whether a manipulation of one variable has an impact on others. And most of the studies focus on relationships between only one or two atmospheric processes with changes in Arctic sea ice. Therefore, in this study, we target to provide a more comprehensive analysis about causality between multiple atmospheric processes and sea ice by applying different data-driven causality approaches.

## 3 Data Sets and Data Pre-processing

In this study, we use the total sea ice extent as the Arctic sea ice index. The sea ice extent is defined as the total area in the Arctic with sea ice concentration greater than 15%. The conversion from sea ice concentration to sea ice extent was conducted at daily time scale. Therefore, we obtained the sea ice concentration from the Nimbus-7 SSMR and DMSP SSM/I-SSMIS passive microwave data version 1 (Cavalieri et al., 1996) provided by the National Snow and Ice Data Center. This dataset was generated from brightness temperature data, and provided daily in the polar stereographic projection with a grid box of 25 km × 25 km since October 1978. The uncertainty of sea ice concentration over the Arctic is within ±5*%* during the winter, when the sea ice is relatively thick and the sea ice concentration is high. During the summer, the uncertainty increases to ±15*%* when the melt ponds are present (Cavalieri et al., 1992).

The atmospheric variables were obtained from ERA-5 global reanalysis product. ERA-5 was produced using 4D-Var data assimilation in CY41R2 of European Centre for Medium-Range Weather Forecasts (ECMWF)’s Integrated Forecast System (IFS), with 137 hybrid sigma/pressure (model) levels in the vertical, with the top level at 0.01 hPa (Hersbach et al., 2020). The ERA-5 reanalysis has been evaluated over the Arctic in the previous studies and it stands out among several global reanalysis products (Graham et al., 2019; Mayer et al., 2019) as being more consistent with independent observations (Hersbach et al., 2020), which lends credence to the results obtained here in connection with the associations between the variables considered. In this study, the variables used in the causality discovery algorithms are listed in Table 1. For three-dimensional data (geopotential heights and relative humidity), we treat it as a single variable because we would like to filter out the connections between different layers for each variable. The air temperature has been excluded in this study because it exhibits very high correlation with sea ice concentration. The interactions between air temperature and sea ice could be dominant over all other atmospheric processes based on our tests.

**TABLE 1:** The atmospheric and sea ice variables considered in this study.

Abbrev.in Figure 1	Variable
GH	Geopotential heights averaged from 200 hPa, 500 hPa, and 850 hPa
RH	Relative humidity averaged from 1,000–300 hPa
SLP	Sea level pressure
u10 m	Zonal (u-component) wind at 10 m
v10 m	Meridional (v-component) wind at 10 m
HFLX	Sensible plus latent heat flux
Precip	Total precipitation
CC	Total cloud cover
CW	Total cloud water path
SW	Net shortwave flux at the surface
LW	Net longwave flux at the surface
Sea_ice	Sea ice extent in the Northern Hemisphere

**FIGURE 2:**
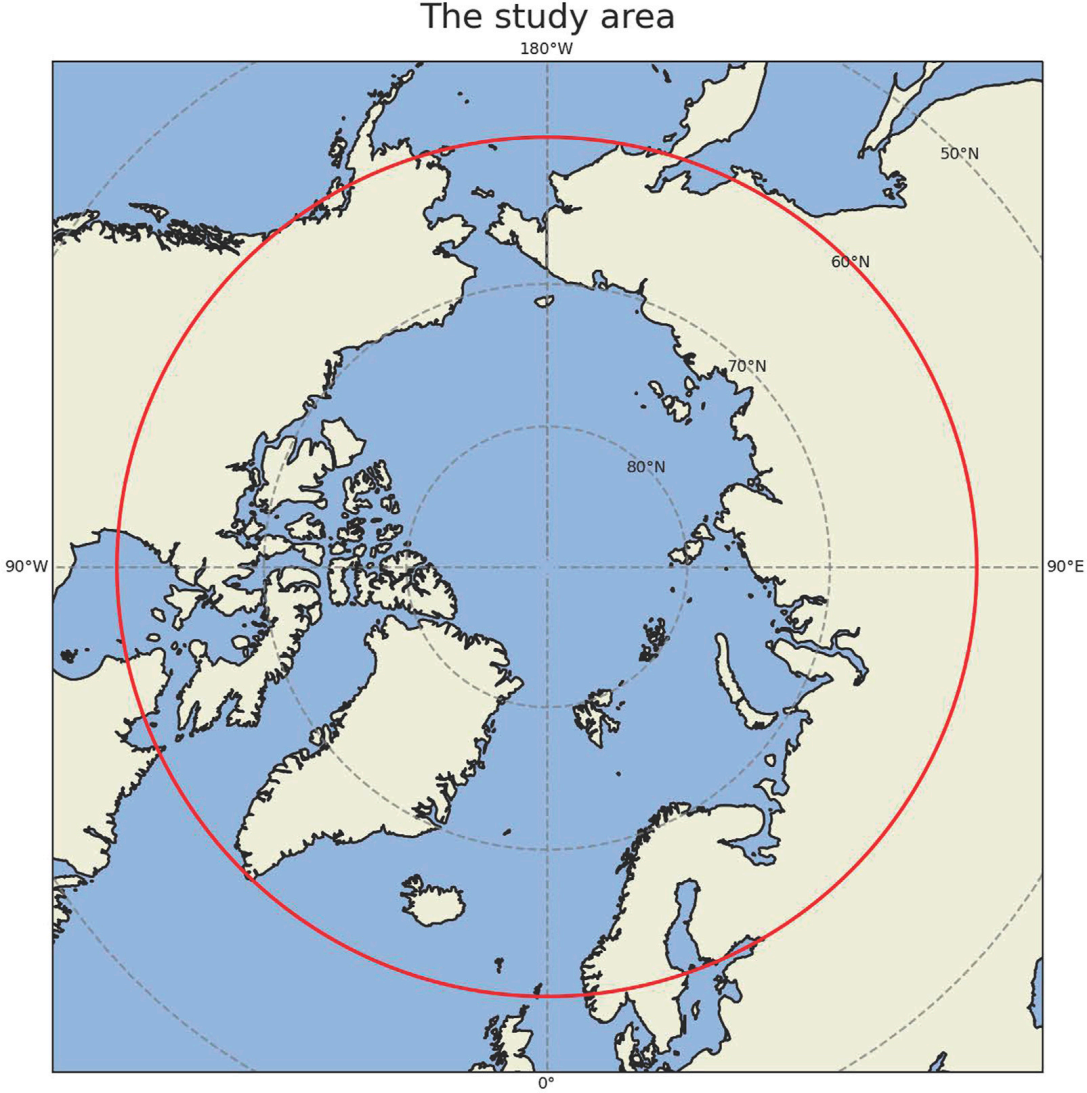
The study area (60°N northward). The circle of 60°N is marked in red. Depending on the season, sea ice could occur south of 60°N.

All monthly gridded data during 1980–2018 have been averaged over the Arctic north of 60°N (Figure 2) using area-weighted method. Therefore, we created the time-series for both sea ice extent and atmospheric variables. We believe that 39 years of data should be sufficient to derive causal relationships and draw meaningful conclusions. In addition, most of the observational-based climate studies mentioned in Figure 1 used the data during the modern satellite era (1979-present), which is consistent with our studies. Our purpose is to match this time period and to determine whether those algorithms can produce similar results. Under the background of global warming, almost all components in the Earth System changed with time, as a response to increased greenhouse gas emissions. Regressing atmospheric responses against sea ice decline (or the other way around) involves the risk of finding potentially spurious atmosphere-sea ice interactions simply because both variables change across years (Iler et al., 2017). Therefore, it is necessary to detrend the time-series as the climate data is normally nonlinear and nonstationary (Wu et al., 2007). This technique has been widely used in previous climate studies (Weber and Talkner, 2001; Kawale et al., 2013), including several recent studies about atmosphere-sea ice interactions in the Arctic (Ding et al., 2017; Huang et al., 2017; Baxter et al., 2019; Topál et al., 2020). Moreover, detrending for time-series that is nonstationary is also important in causality discovery methods (Granger, 1969; Entner and Hoyer, 2010; Peters et al., 2012; Runge et al., 2019). Thus, in order to eliminate overall impacts of global warming and seasonality during this 39-year time period, we applied detrending and deseasonalizing for each time-series. Note that we also conducted additional analysis with raw data to show how detrending and deseasonalizing have an impact on our results at the end of Section 5.

Here we assume the time series is additive and there exist both trend and seasonal components, that is

$$X_{t}=m_{t}+s_{t}+Y_{t}.$$ (1)

Here, the *m*_*t*_ indicates the trend component, while the *s*_*t*_ represents seasonality component. The time series has been detrended by subtracting the line of best fit from the time-series *m*_*t*_, where the line of best fit was obtained from a linear regression model with the time steps as the predictor. To deseasonalize the time-series, we used averaged seasonal index *s*_*t*_ to seasonally adjust the data. The seasonal index were calculated from moving averages with a 12-months seasonal window in this study (Hamilton, 1994). More details about time series decomposition can be found in Brockwell et al. (2016). At the end, we only kept the residual component *Y*_*t*_, which fluctuates around zero, that is

$$E\left(Y_{t}\right)=0.$$ (2)

Then we normalized *Y*_*t*_ using the max-min method so that

$$Y_{t}\in \left[0,1\right].$$ (3)

## 4 Data-Driven Causality Discovery Algorithms

**FIGURE 3:**
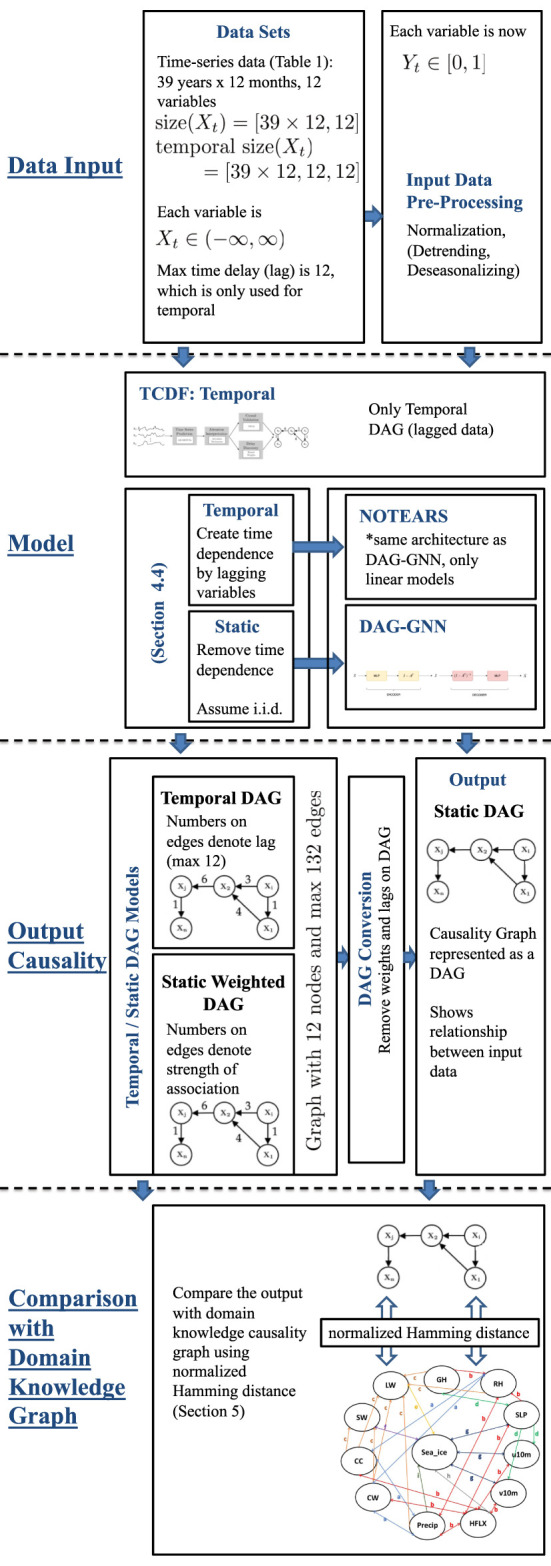
The framework of benchmarking causality discovery methods in atmosphere-sea ice study. Note that detrending and deseasonalizing time-series is optional during the input data pre-processing process.

In this work, we apply data-driven causal discovery algorithms aiming to find the major causes of the decrease of Arctic sea ice. These algorithms typically assume one process or state, a cause, contributes to the production of another process or state, an effect. The cause is assumed to be partly responsible for the effect, and the effect is partly dependent on the cause. Although it is not necessary that the effect will have a reverse *affect* on the cause. Thus, causal discovery aims to discover direct cause-effect relationships for both instantaneous and delayed causes. Here we will investigate three recently proposed causal discovery algorithms: TCDF (Nauta et al., 2019), NOTEARS (Zheng et al., 2018), and DAG-GNN (Yu et al., 2019). The overall framework of our benchmarking pipeline is shown in Figure 3. We believe this general framework can help researchers to evaluate their causality discovery approaches in sea ice study and Earth science in general.

### 4.1 Temporal Causality Discovery Framework

**FIGURE 4:**
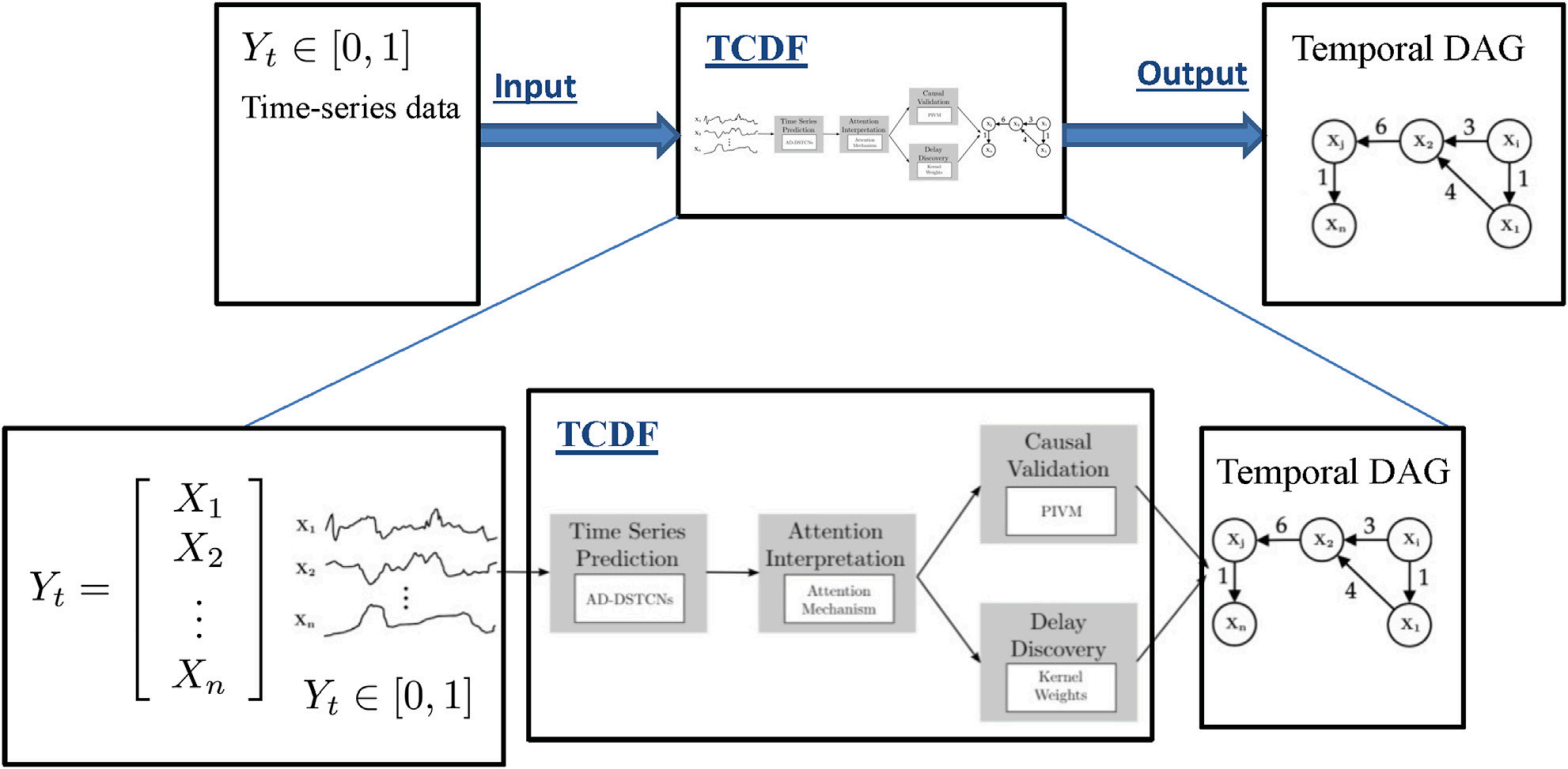
Architecture of TCDF (Nauta et al., 2019). The figure is an illustrative example of a high level view of how TCDF creates a causal discovery graph with delays (numbers on the edges of the graph) from time-series data.

The TCDF algorithm (Nauta et al., 2019) is based on attention-based (Yin et al., 2016) Convolutional Neural Networks (CNN). The input to the algorithm is time series data and the output is a causality graph structure with time delay or lag, which is automatically determined by the TCDF algorithm. For our climate sciences problem with data shown in Table 1, the TCDF algorithm takes the measured data of sea ice, relative humidity, and other atmospheric processes (Table 1) in order to build a causality graph for the input data interact with each other. Figure 4 illustrates the architecture of the TCDF method, operating on generic data, where the multi-dimensional time-series data is on the left and the produced causality graph is on the right. There are four steps to learn a Temporal Causal Graph from the time-series data: Time Series Prediction block, Attention Interpretation block, Causal Validation and Delay Discovery blocks as explained in detail in Nauta et al. (2019). Here we explain the general process of the TCDF algorithm where a more in depth description is in section 4 of Nauta et al. (2019). The first step is that the time-series data is fed into the Time Series Prediction block, which tries to create an internal time-series model that will accurately try to model each atmospheric or atmosphere-sea ice process. Then the Attention Interpretation block takes that hidden model produced by the Time Series Prediction block and tries to verify and validate how accurate the prediction is to the actual data. Then the last two blocks again verify the hidden model from the Time Series Prediction block but now using the verification errors from the Attention Interpretation block in parallel. The two last blocks try to verify the causal and time delay relationships generated from the prediction block with errors generated by the attention block. A detailed explanation is provided in Nauta et al. (2019) and more details on attention-based CNN can be found in Yin et al. (2016). Also as a side note: for multi-dimensional time-series there are *n* independent attention-based CNNs, all with the same architecture for each time-series data.

The basic structure of TCDF is for time-series prediction as seen in the first step of the framework in Figure 4. After predicting time-series, the output gives attention scores for the attention interpretation mechanism. Attention CNNs (Yin et al., 2016) is a machine learning method based on using neural networks to help optimize internal automatically picked parameters in the hidden model generated in our case from the Time Series block. In other words, it is a form of self-learning or adaptive optimization (Eveleigh, 1967; Wei, 2018) applied to machine learning. The causality validation reads the final result of the attention scores and applies a permutation importance validation method. The permutation importance is a measurement of how much an error will affect the values of a certain attention score when all scores are randomly permuted. The idea is that permuting a time-series attention score removes potential cause and effect relationships and hence the method can detect real versus fake causal relationships. In parallel the attention scores are fed to the delay discovery to learn the potential delay in cause and effect relationships. The delay discovery also employs the permutation importance validation method.

Another major advantage of TCDF is in using a CNN versus a traditional Recurrent Neural Network (RNN), such as a Long Short Term Memory (LSTM), for time-series data. The advantage is that RNNs typically have a vanishing gradient problem: long-term information has to sequentially travel through all the cells before getting to the present processing cell and typically stalls the learning processes, sometimes even preventing any further improvements gained with learning with more data (Hochreiter, 1998; Tan and Lim, 2019). This is greatly amplified when the number of layers becomes very deep, typically more than 10 layers (Schmidhuber, 2015). Though a CNN structure might have this problem as well, it is more common in RNN because it typically needs much more memory and cells than a CNN structure. With more cells to process, there is a greater chance of obtaining the vanishing gradient problem.

### 4.2 Non-combinatorial Optimization *via* Trace Exponential and Augmented lagrangian for Structure learning

The NOTEARS algorithm (Zheng et al., 2018) assumes a linear data generating model of the form

$$X_{i}=\underset{j:W_{ji}\neq 0}{\sum }W_{ji}X_{j}+N_{i},$$ (4)

where *W* is the weighted adjacency matrix of the underlying causality graph *G*(*W*), that is *j* → *i* in *G*(*W*) if and only if *W*_*ji*_ ≠ 0, and the random variables *N*_*i*_ are independent noise variables. Given *n* independent and identically distributed (i.i.d.) observations of the variables *X*_1_, … , *X*_*d*_, written as matrix **X** ∈^*n*×*d*^, a standard estimator for *W* is the (regularized) least-squares estimator

$$\hat{W}=arg\underset{W\in^{d\times d}}{\;\min}\frac{1}{2n}\|X-XW{\|}_{F}^{2}+\lambda \|W{\|}_{1}\;subjecttoG\left(W\right)isaDAG,$$ (5)

where *λ* ≥ 0 is the regularization parameter. This estimator is theoretically well-studied and satisfies desirable properties such as consistency (Van de Geer and Bühlmann, 2013; Loh and Bühlmann, 2014; Aragam et al., 2017). However, due to the non-convex, *combinatorial-like* constraint, optimization problems of the form Eq. 5 are NP-hard to solve (Chickering, 1996), and hence unless the number of variables *d* is very small, heuristics such as local search have to be applied (e.g., Heckerman et al., 1995; Ramsey et al., 2017). The NOTEARS algorithm builds on the insight that

G(W) is a DAG⇔trace(exp(W◦W))−d=0, (6)

where exp denotes the matrix exponential and ◦ the element-wise product. The characterization (6) allows to treat the optimization problem Eq. 5 as an ordinary *continuous* constrained optimization problem and to use any algorithm from the rich literature on continuous optimization to find a locally optimal solution to (5). Concretely, the NOTEARS algorithm applies the augmented Lagrangian method (e.g., Nocedal and Wright, 2006) to search for a locally optimal solution to

$$arg\underset{W\in^{d\times d}}{\min}\frac{1}{2n}\|X-XW{\|}_{F}^{2}+\lambda \|W{\|}_{1}\;\text{subject to trace }\left(\exp\left(W◦W\right)\right)-d=0.$$ (7)

After applying the augmented Lagrangian method to (7) and obtaining an output $\tilde{W}$, the final step of the NOTEARS algorithm is to “round” $\tilde{W}$ and to set all entries of $\tilde{W}$ with absolute value smaller than some threshold *t* to zero. This yields the final output W^^ of the NOTEARS algorithm.

In summary, the NOTEARS algorithm yields an estimate of the underlying causality graph as well as the strengths of the causal relationships. It does so by assuming a linear data generating model and access to i.i.d. observations and fitting a causal graph to the data. Its advantage over existing approaches is that it formulates the fitting problem in a way that makes it amenable to standard algorithms for continuous optimization. However, in general, the NOTEARS algorithm will still return only a locally optimal solution to the fitting problem, and the assumption of a linear data generating model might restricts its applicability.

### 4.3 Directed Acyclic Graph-Graph Neural Networks

DAG-GNN (Yu et al., 2019) can be thought of as an extension to the NOTEARS algorithm (Zheng et al., 2018) in that the proposed method assumes a nonlinear model of the form

$$X=f_{2}\left({\left(I-A^{T}\right)}^{-1}f_{1}\left(Z\right)\right),$$ (8)

where *Z* is the encoded latent variable of *X*. This can be contrasted to the linear model assumed in NOTEARS

$$X={\left(I-A^{T}\right)}^{-1}Z,$$ (9)

where Eq. 9 is a restructured form of Eq. 4. Further, DAG-GNN builds an inference model to encode *Z*, given by

$$Z=f_{4}\left(\left(I-A^{T}\right)f_{3}\left(X\right)\right),$$ (10)

where *f*_3_ and *f*_4_ play a conceptually inverse role for *f*_2_ and *f*_1_ respectively. In particular, this paper assumes *f*_1_, *f*_4_ to be identity functions and *f*_2_, *f*_3_ as Multilayer Perceptrons (MLP). Multilayer Perceptrons are feed-forward Artificial Neural Networks with multiple hidden layers. They are trained through stochastic gradient descent and backpropagation and their function (*f*_2_ and *f*_3_ in our case) corresponds to the relation between the input and output variables.

**FIGURE 5:**
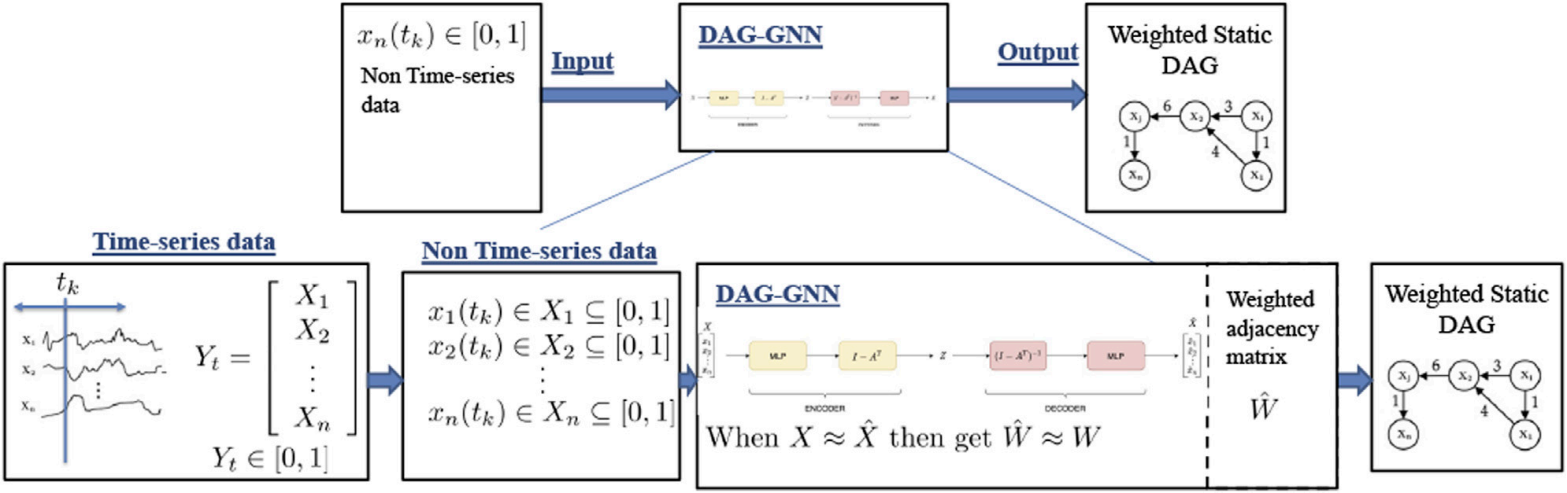
Architecture of DAG-GNN (Yu et al., 2019). *X* is the observed data and X^^ its reconstruction, which is sampled from a factored Gaussian with mean *M*_*X*_ and standard deviation *S*_*X*_.

Since an MLP is nonlinear, it should in theory capture any nonlinearities in the data better than NOTEARS which is a linear model. This is further explained in Figure 5 where $\hat{X}$ is the regenerated form of *X* and MLP has one hidden layer of 64 neurons. Further, DAG-GNN minimizes the following loss function.

$$\begin{array}{ll} \underset{A,\theta }{\min} & f\left(A,\theta \right)=-L_{ELBO} \end{array}$$ (11)

$$\begin{array}{ll} \text{s.t. } & h\left(A\right)=\text{tr}\left[{\left(I+\alpha A◦A\right)}^{m}\right]-m=0, \end{array}$$ (12)

where the unknowns include the weight matrix *A*, and parameters *θ* for Variational Autoencoder (VAE). Further, ELBO is the Evidence Lower Bound of the VAE adopted from (Kingma and Welling, 2014) and *h*(*A*) is used to solve the augmented Lagrangian as done in (Nocedal and Wright, 2006).

So DAG-GNN is a more robust, non-linear model which can learn more complex relationships than NOTEARS. Having an MLP as a backbone it is capable of learning from a large training set, and giving accurate results. Furthermore, autoencoders have been proven to be useful for unsupervised learning and feature extraction. This makes DAG-GNN capable for understanding causal relationships between atmospheric variables.

### 4.4 Static Versus Temporal Model

While TCDF requires time series data as input and explicitly models time delay of causal relations, NOTEARS and DAG-GNN assume to be provided i.i.d. observations of the variables. Similarly to other causal discovery studies in climate research (Ebert-Uphoff and Deng, 2012), we apply the latter two methods in two ways: in the *static model*, we treat the observations of the variables summarized in Table 1 at different points in time as i.i.d. observations and directly feed the data into the two methods. Alternatively, in the *temporal model*, we first augment the data set by adding lagged versions of each variable, that is for each variable *X* we additionally consider variables *X* ^1^, *X* ^2^, … , *X* ^12^, where *X* ^*k*^ is a version of the variable *X* that is measured with a lag of *k* time units (in our case: months) compared to *X*. Here, the maximum time lag we consider is 12 months since we want to focus on causal links from sub-seasonal to seasonal timescales. We then treat the observations of the various variables at different points in time as i.i.d. observations and run NOTEARS and DAG-GNN, respectively. The graphs produced by these methods, using the augmented data, are assumed to encode the time delay of causal relations. However, in order to obtain a causal graph on the variables of Table 1 we generate a “reduced” temporal graph from these “full” temporal graphs by connecting two variables *X* and *Y* in the reduced temporal graph whenever any of the variables *X*, *X* ^1^, … , *X* ^12^ is connected to any of *Y*, *Y* ^1^, … , *Y* ^12^ in the full temporal graph. The reduced temporal graph is the output of the temporal model.

## 5 Results

In this section we present some results of the three causal discovery algorithms introduced in Section 4. We study how the causality graphs produced by the three methods depend on the choice of hyperparameters and see that the graphs can be quite different for varying hyperparameters. We work with the normalized Hamming distance (ignoring the edge weights in the graphs produced by NOTEARS or DAG-GNN) and also compare all graphs to the domain knowledge graph (Figure 1). Note that We do not quantify the strength of causal relationships in the domain knowledge graph, and we evaluate the algorithms in terms of whether they are capable of detecting these causal relationships, but not in terms of estimating the strength of relationships.

In this section, we treat all graphs as unweighted graphs. The normalized Hamming distance is a widely used metric to compare two unweighted graphs on the same set of vertices (Donnat and Holmes, 2018). Let *A*, *B* ∈{0,1}^*m*×*m*^ be the adjacency matrices of two unweighted graphs *G*_*A*_, *G*_*B*_ on *m* vertices. The normalized Hamming distance between *G*_*A*_ and *G*_*B*_ is given by $dist_{HD}\left(G_{A},G_{B}\right)=\frac{1}{m^{2}}\sum_{i,j=1}^{m}1\left\{A_{ij}\neq B_{ij}\right\}$, that is the number of edges that are present in one graph but not in the other, normalized by the number of all possible edges. The normalized Hamming distance between *G*_*A*_ and *G*_*B*_ is zero if and only if *G*_*A*_ and *G*_*B*_ coincide, and it is at most one (which happens if one graph is empty, i.e., does not have any edges, and one graph is complete, i.e., any two vertices are connected). In the following, for each of the three causal discovery algorithms introduced in Section 4 we compute the normalized Hamming distance between the graphs produced by an algorithm for different values of its hyperparameters. We also compare the graphs to the domain knowledge graph shown in Figure 1 which is generated based on current literature.

### 5.1 Causality Discovery Results Based on Temporal Causality Discovery Framework Approach

Table 2 shows the values for the normalized Hamming distance for the TCDF method, which quantifies the similarity of two causality graphs. A smaller number indicates that two causality graphs are more similar to each other. Two hyperparameters that were chosen are the kernel size, which is how many data points are combined together, and the number of hidden layers, which perform nonlinear transformations of the inputs entered into the network. The number of hidden layers corresponds to the number of hidden CNN layers in the TCDF algorithm. It seems that the addition of a hidden layer leads to far worse results and even produces no causality graphs as is the case for when *kernel* = 4 and *kernel* = 6 for *layer* = 1. The kernel size is related to how much the TCDF method lags the variables for the causality study. The default setting for the hyperparameters as shown with *♣* in Table 2 produce TCDF’s best result when comparing to the domain knowledge graph, but that is nowhere close to the domain knowledge graph shown in Figure 1. It seems that the TCDF method does not produce good results for our Arctic Sea Ice data.

**TABLE 2:** Distance matrix with respect to the normalized Hamming distance for TCDF. *♣* denotes *layer* =0, *kernel* =4 are the algorithm’s default hyperparameters. The bottom row compares to the domain knowledge graph of Figure 1, with the best values being marked in bold.

		*Temporal*					
		*layer* = 0	*layer* = 0	*layer* = 0	*layer* = 1	*layer* = 1	*layer* = 1
		*kernel* = 2	*kernel* = 4^*♣*^	*kernel* = 6	*kernel* = 2	*kernel* = 4	*kernel* = 6
*Temporal*	*layer* = 0, *kernel* = 2	0	0.05	0.01	0.02	0.01	0.01
	*layer* = 0, *kernel* = 4^*♣*^	0.05	0	0.06	0.07	0.06	0.06
	*layer* = 0, *kernel* = 6	0.01	0.06	0	0.01	0.01	0.01
	*layer* = 1, *kernel* = 2	0.02	0.07	0.01	0	0.02	0.02
	*layer* = 1, *kernel* = 4	0.01	0.06	0.01	0.02	0	0
	*layer* = 1, *kernel* = 6	0.01	0.06	0.01	0.02	0	0
	Domain knowl	0.35	**0.33**	0.34	0.34	**0.33**	**0.33**

#### 5.1.1 Comparison Between Temporal Causality Discovery Framework Based Causality Graph and Domain Knowledge Graph

**FIGURE 6:**
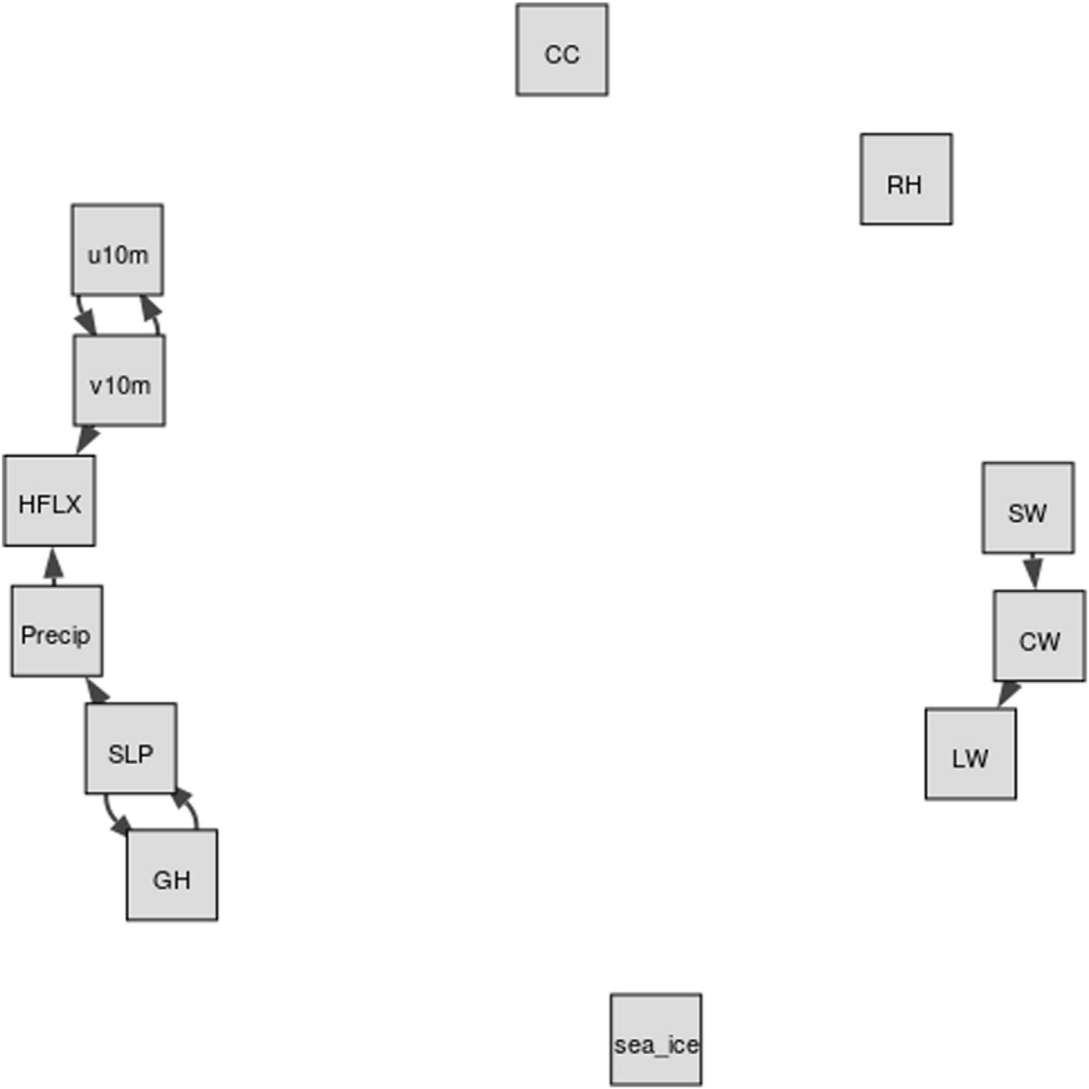
The TCDF graph that is closest to the domain knowledge graph of Figure 1. The temporal graph for *layer* =0, *kernel* =4.

Since the TCDF focuses on time series, only the temporal graph that is closest to the domain knowledge graph is shown (Figure 6). In general, if the causality graph generated by the algorithm looks similar to Figure 1, we believe that this approach is more capable of capturing the real causal relationships in the Arctic. There is no cause and effect between sea ice and any atmospheric variables. As for the causality within the atmosphere, only a few edges (cause-effect relationships) are generated by the TCDF algorithm. Among them, the feedback between u10 m and v10 m, as well as the impact of SW on CW are not consistent with domain knowledge.

### 5.2 Causality Discovery Results Based on Non-combinatorial Optimization *via* Trace Exponential and Augmented lagrangian for Structure learning Approach

The NOTEARS algorithm has two hyperparameters *λ* ≥ 0 and *t* ≥ 0 as explained in Section 4.2: the parameter *λ* is the regularization parameter (cf. Eq. 5) and *t* is the threshold for setting edge weights of the preliminary output to zero (cf. end of Section 4.2). There is no default value for *λ*, but in the main experiment that comes with the NOTEARS code (Zheng et al., 2018), the authors use *λ* = 0.1 and hence we consider that value to be the default value. Furthermore, we observed that choosing a value larger than 0.1 for *λ* often results in an empty graph as the output of NOTEARS. The default value for *t* is *t* = 0.3. Indeed, we observed that *t* = 0.3 yields better results when comparing to the domain knowledge graph than other values of *t*.

Table 3 shows the normalized Hamming distance between the graphs produced by NOTEARS for *λ* ∈{0, 0.1} and *t* ∈{0.2, 0.3}, for both the static and the temporal model. The last row of the table shows the normalized Hamming distance between the various graphs and the domain knowledge graph of Figure 1. Note that actually none of the graphs considered here is a DAG. We can see that the distances between the various temporal graphs (middle right part of the table) are significantly larger than the distances between the various static graphs (upper left part of the table). We can also see that changing the value of *λ* from 0 to 0.1 causes a larger difference in the result than changing the value of *t* from 0.2 to 0.3 (e.g., the normalized Hamming distance between the static model with *λ* = 0, *t* = 0.2 and the static model with *λ* = 0, *t* = 0.3 is only 0.02, while the distance between the static model with *λ* = 0, *t* = 0.2 and the static model with *λ* = 0.1, *t* = 0.2 is 0.15).

**TABLE 3:** Distance matrix with respect to the normalized Hamming distance for NOTEARS. *♣* denotes that *λ* =0.1, *t* =0.3 are the algorithm’s default hyperparameters. The bottom row compares to the domain knowledge graph of Figure 1, with the best values being marked in bold.

		*Static*				*Temporal*			
		*λ* = 0	*λ* = 0	*λ* = 0.1	*λ* = 0.1	*λ* = 0	*λ* = 0	*λ* = 0.1	*λ* = 0.1
		*t* = 0.2	*t* = 0.3	*t* = 0.2	*t* = 0.3^*♣*^	*t* = 0.2	*t* = 0.3	*t* = 0.2	*t* = 0.3^*♣*^
*Static*	*λ* = 0, *t* = 0.2	0.0	0.02	0.15	0.15	0.54	0.36	0.16	0.15
	*λ* = 0, *t* = 0.3	0.02	0.0	0.15	0.12	0.53	0.35	0.14	0.12
	*λ* = 0.1, *t* = 0.2	0.15	0.15	0.0	0.02	0.51	0.36	0.09	0.1
	*λ* = 0.1, *t* = 0.3^*♣*^	0.15	0.12	0.02	0.0	0.52	0.35	0.07	0.08
*Temporal*	*λ* = 0, *t* = 0.2	0.54	0.53	0.51	0.52	0.0	0.18	0.48	0.51
	*λ* = 0, *t* = 0.3	0.36	0.35	0.36	0.35	0.18	0.0	0.33	0.34
	*λ* = 0.1, *t* = 0.2	0.16	0.14	0.09	0.07	0.48	0.33	0.0	0.03
	*λ* = 0.1, *t* = 0.3^*♣*^	0.15	0.12	0.1	0.08	0.51	0.34	0.03	0.0
	Domain knowl.	0.35	**0.33**	0.36	0.35	0.54	0.46	0.37	**0.35**

#### 5.2.1 Comparison Between Non-combinatorial Optimization *via* Trace Exponential and Augmented lagrangian for Structure learning Based Causality Graph and Domain Knowledge Graph

**FIGURE 7:**
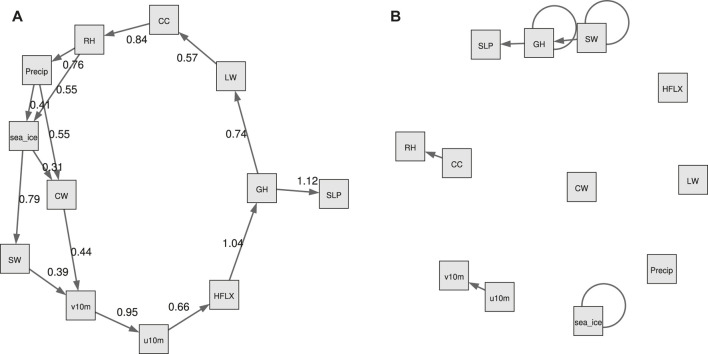
The NOTEARS graphs that are closest to the domain knowledge graph of Figure 1, with respect to the normalized Hamming distance as shown in Table 3. **(A)**: The static graph for *λ* = 0, *t* = 0.3. Note that in Table 3 this graph is treated as an unweighted graph.The edge weights are estimates of the coefficients *W*_*ji*_ in the data generating model (4). The larger weights indicate stronger connection between two variables. **(B)**: The temporal graph for *λ* = 0.1, *t* = 0.3.

In Figure 7, we show both the static and the temporal graphs that are closest to the domain knowledge graph of Figure 1. The larger weights in the causality graph indicate a stronger relationship between two variables. Generally, while none of the produced graphs is really close to the domain knowledge graph, the static graph looks more reasonable.

In the static graph, the RH and precipitation seem to dominate the sea ice changes, with weights of 0.55 and 0.41, respectively. In the meantime, the sea ice exerts large influence on SW (weight of 0.79) and CW (weight of 0.31). The causal relations between precipitation, SW and sea ice in the domain knowledge graph of Figure 1 are well captured by the NOTEARS algorithm. However, RH and CW are believed to be only indirectly connected with sea ice changes (i.e., in the domain knowledge graph there is no direct connection between RH or CW and sea ice), but in the static graph produced by the NOTEARS algorithm there are direct connections. The causality between each two atmospheric variables is generally reasonable based on the domain knowledge graph. The connections between CW and v10 m, SW and v10 m, and v10 m and u10 m are not quite consistent with the domain knowledge graph. However, those connections may be physically reasonable because winds are related to changing temperatures, humidity, clouds and radiation through advection in a broader area. Compared with the static graph, the temporal graph detects only very few edges. It shows that the sea ice, SW and GH have delayed impacts on themselves, demonstrating both sea ice and atmosphere have a degree of seasonal to year long climate predictability. Note that the NOTEARS does not model time delay of causal relations as mentioned in Section 4.4 and the temporal graphs that we produced using this algorithm do not contain straightforward-to-interpret time lag information.

### 5.3 Causality Discovery Results Based on Directed Acyclic Graph-Graph Neural Networks Approach

Like the NOTEARS algorithm, DAG-GNN has two hyperparameters: *τ* ≥ 0 and *t* ≥ 0, where *τ* is similar to *λ* used in NOTEARS. We noticed that the hyperparameter *τ* is very sensitive, and show the outputs for only two values of *τ* i.e., *τ* ∈{0, *e* − 7}. We vary *t* similar to NOTEARS, and test for *t* ∈{0.2, 0.3}. Table 4 tabulates the normalized Hamming Distance computed between all the graphs obtained by varying these two hyperparameters. Further, we computed the Normalized Hamming Distance between all these graphs and the domain knowledge graph of Figure 1. In order to carry out this specific computation, we created unweighted matrices from the weighted outputs of DAG-GNN with the help of absolute thresholding using the hyperparameter *t*.

**TABLE 4:** Distance matrix with respect to the normalized Hamming distance for DAG-GNN. *♣* denotes the algorithm’s default hyperparameters. The bottom row compares to the domain knowledge graph of Figure 1, with the best values being marked in bold.

		*Static*				*Temporal*			
		*τ* = 0	*τ* = *e* − 7	*τ* = 0	*τ* = *e* − 7				
		*t* = 0.2	*t* = 0.3^*♣*^	*t* = 0.2	*t* = 0.3	*t* = 0.2	*t* = 0.3^*♣*^	*t* = 0.2	*t* = 0.3
*Static*	*τ* = 0, *t* = 0.2	0.0	0.06	0.04	0.07	0.1	0.12	0.1	0.12
	*τ* = 0, *t* = 0.3^*♣*^	0.06	0.0	0.05	0.01	0.08	0.07	0.08	0.07
	*τ* = *e* − 7, *t* = 0.2	0.04	0.05	0.0	0.06	0.08	0.1	0.08	0.1
	*τ* = *e* − 7, *t* = 0.3	0.07	0.01	0.06	0.0	0.08	0.08	0.08	0.08
*Temporal*	*τ* = 0, *t* = 0.2	0.1	0.08	0.08	0.08	0.0	0.03	0.01	0.03
	*τ* = 0, *t* = 0.3^*♣*^	0.12	0.07	0.1	0.08	0.03	0.0	0.05	0.0
	*τ* = *e* − 7, *t* = 0.2	0.1	0.08	0.08	0.08	0.01	0.05	0.0	0.05
	*τ* = *e* − 7, *t* = 0.3	0.12	0.07	0.1	0.08	0.03	0.0	0.05	0.0
	Domain knowl	0.33	0.33	0.35	**0.32**	0.35	**0.34**	0.36	**0.34**

**FIGURE 8:**
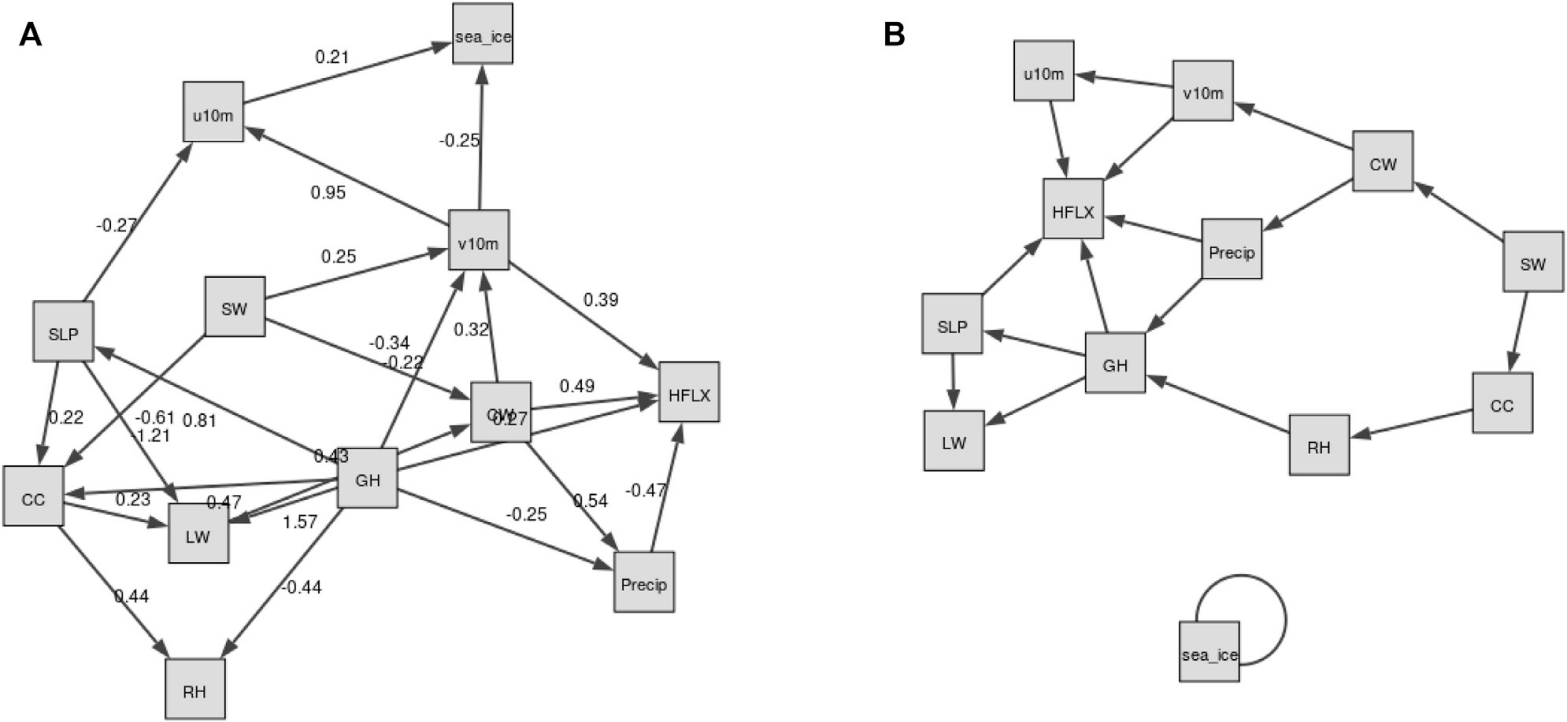
The DAG-GNN graphs that are closest to the domain knowledge graph of Figure 1. **(A)**: The static graph for *τ* = 0, *t* = 0.2. The larger weights indicate stronger connection between two variables. **(B)**: The temporal graph for *τ* ∈{0, *e* − 7}, *t* = 0.3.

From Table 4, we see that the least normalized Hamming Distance with the Domain Knowledge Graph is obtained by *τ* = *e* − 7, *t* = 0.3 for the static model and *τ* ∈{0, *e* − 7}, *t* = 0.3 for the temporal model. Both the temporal models of *t* = 0.3 give the same graphs, which is shown in Figure 8 on the right. For the static model however, these optimum values of *τ* and *t* produce a graph which shows no relation between sea ice and other atmospheric variables. Hence, we reject it. The second most optimum graph showing a dependence of sea ice with atmospheric variables is shown in Figure 8 on the left. Its hyperparameters are *τ* = 0 and *t* = 0.2. Further, we note that for the temporal model, the best graphs are obtained with *t* = 0.3, which is one of the default parameters used and suggested by the authors in (Yu et al., 2019).

#### 5.3.1 Comparison Between Directed Acyclic Graph-Graph Neural Networks Based Causality Graph and Domain Knowledge Graph

The static and the temporal graphs closest with the domain knowledge graph are shown in Figure 8. Compared to NOTEARS, both static and temporal graphs produced by DAG-GNN are more complicated. The dynamical fields (u10 m and v10 m) dominate the sea ice changes, but with relatively small weights 0.21 and −0.25, respectively. Here, the positive (negative) edge weights indicate a positive (negative) causal effect. In this case, the negative weights between v10 m and sea ice suggest that increasing v10 m tends to decrease sea ice. Stronger northward winds can enhance ice melt by increasing ice drifting (Spreen et al., 2011) and bringing more heat and moisture from lower latitudes (Zhang et al., 2013). In the meantime, positive zonal winds (u10 m) generally isolate the Arctic from mid-latitudes, leading to cooling (in winter) and thus more sea ice (Overland and Wang, 2010). As for the causal relations between multiple atmospheric processes, they are generally reasonable compared to Figure 1. Similar as NOTEARS, the connections between u10 m and v10 m (0.95), SW and v10 m (0.25), CW and v10 m (0.32) are not reasonable and consistent with domain knowledge graph. As mentioned earlier, this is most likely because we averaged these variables in the Arctic region and those variables may exhibit relatively high correlations over a large domain. Note that the increased CW and CC tend to reflect solar radiation back to the space, leaving less SW reaching at the surface. This negative relationships between CC, CW, and SW are captured by the DAG-GNN, however, the direction of arrows are not meaningful. The same issue occurs in the temporal graph. In addition, the sea ice has the delayed impacts on itself, but with no connection with any atmospheric processes in temporal graph. Like static graph, the causality between u10 m and v10 m as well as CW and v10 m is not consistent with domain knowledge graph. Similar as NOTEARS, DAG-GNN does not model time delay of causal relations as well.

### 5.4 Sensitivity Tests

**FIGURE 9:**
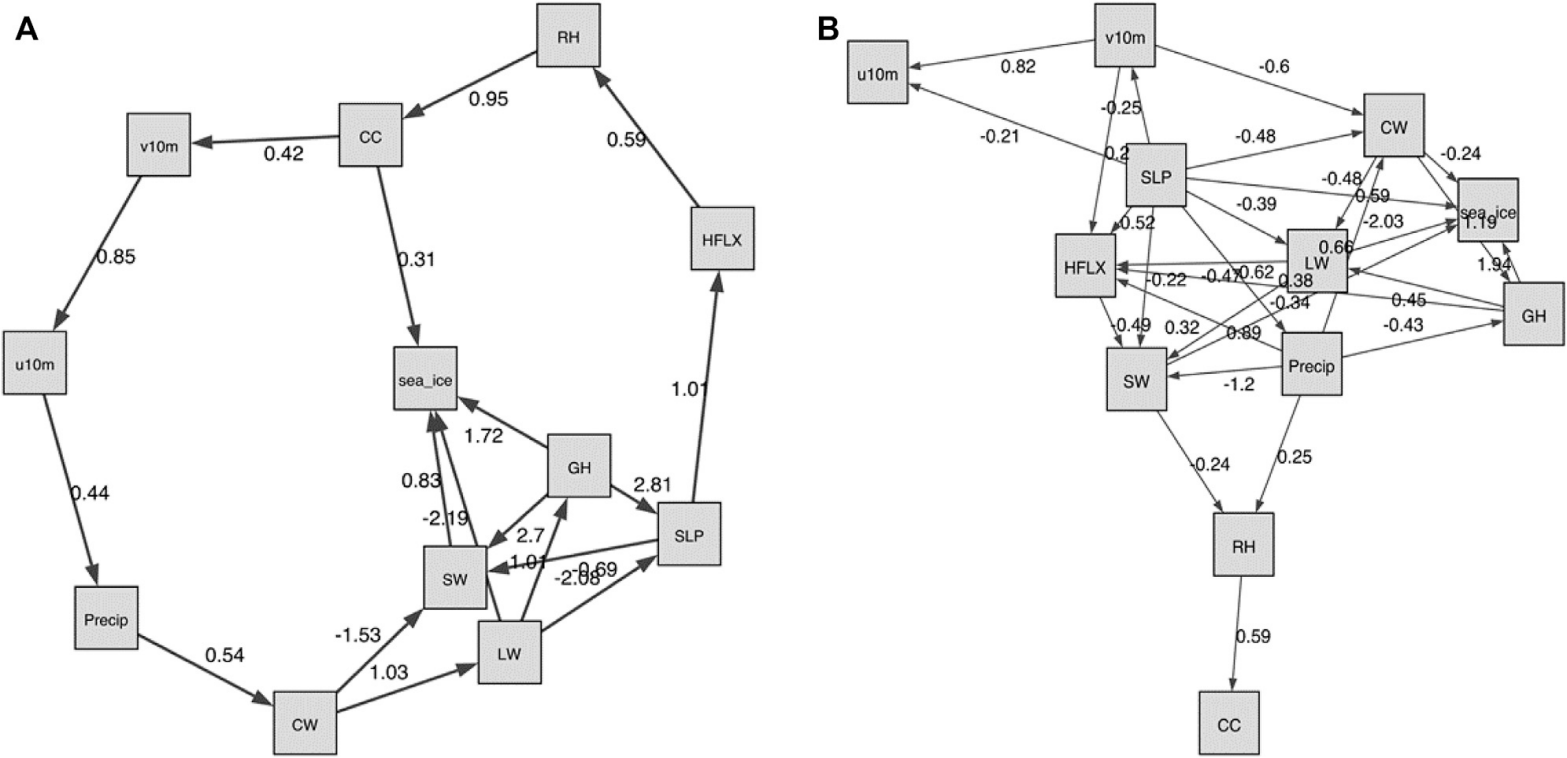
The graphs produced with raw data sets (non-detrended and non-deseasonalized). **(A)**: The NOTEARS static graph for *λ* = 0, *t* = 0.3. **(B)**: The DAG-GNN static graph for *τ* = 0, *t* = 0.2.

We conducted additional tests with slightly different datasets to show how the causality discovery approaches are sensitive to the data. Because the results above are based on the detrended and deseasonalized data sets, the first sensitivity test we did is with raw data. Here, we show the similar static graphs as Figures 7, 8, but with raw data in Figure 9. The TCDF still does not generate meaningful edges between atmospheric components and sea ice (not shown). The NOTEARS produces different results between raw data and detrended data. Using raw data, CC, GH, LW and SW dominate sea ice changes. In comparison, RH and precipitation have a large influence on sea ice variations based on detrended and deseasonalized data. The DAG-GNN also generates more complicated results with raw data. In particular, CW, GH, SLP, LW, and SW are found to dominate sea ice variations, which show similar results as NOTEARS. Note that both NOTEARS and DAG-GNN cannot capture the response in atmosphere to sea ice variations using raw data. As mentioned earlier, the relationships obtained from detrended and deseasonalized data represent natural variability, while raw data provides information about actual changes.

**FIGURE 10:**
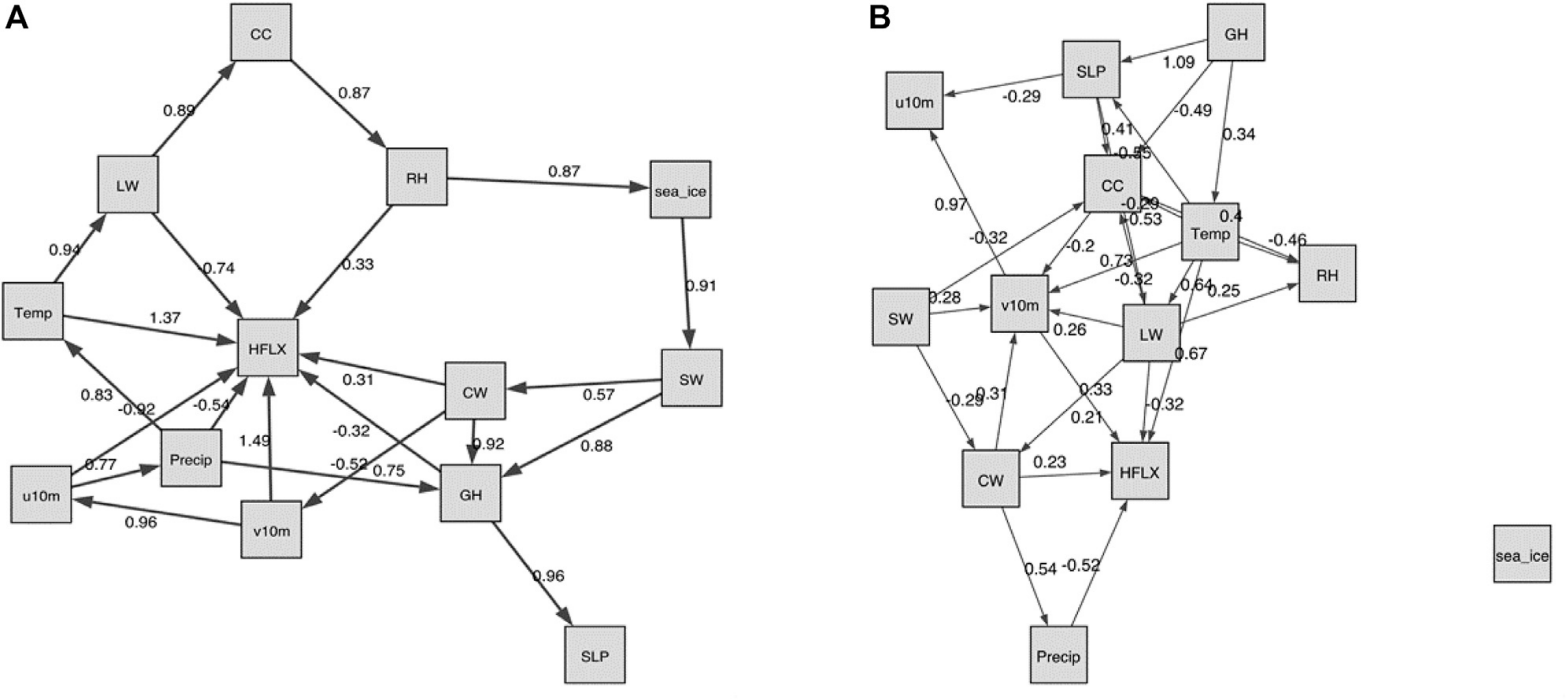
The graphs produced with data sets including air temperature averaged from 1,000 to 300 hPa. **(A)**: The NOTEARS static graph for *λ* = 0, *t* = 0.3. **(B)**: The DAG-GNN static graph for *τ* = 0, *t* = 0.2.

The second sensitivity test we carried out is with dataset that also includes air temperature (averaged from 1,000–300 hPa). In Figure 10, we show the similar static graphs as Figures 7, 8, but include variable “Temp”. In general, the TCDF does not generate any meaningful results (not shown). The NOTEARS shows that temperatures have a large impact on the LW and HFLX, which is physically meaningful based on Stefan-Boltzmann law. In this case, RH dominates the changes in sea ice, while sea ice exerts large influence on SW. These results are similar to Figure 7, in which we excluded temperature. According to results produced by DAG-GNN, temperatures have an impact on SLP, HFLX, CC, RH, LW, and v10 m. These connections are relatively reasonable since we average those variables in a large domain. However, there is no edge between sea ice and atmospheric components anymore. We believe that the connections between sea ice and atmosphere could have been filtered out, because the edges among atmospheric components have much larger weights.

## 6 Conclusion and Discussion

The Arctic has undergone dramatic changes in the past few decades, and sea ice decline is believed to be a key driver for the Arctic amplification. On the one hand, the sea ice is melted by mixed effects of atmospheric dynamical and thermodynamical processes. These processes on the other hand, can be largely affected by sea ice melt. Therefore, this study investigates the causality between multiple atmospheric processes and sea ice variations using three data-driven causality discovery approaches (TCDF, NOTEARS and DAG-GNN). As shown in previous sections, one advantage of utilizing these approaches is that they not only generate causal graphs, but also provide quantified information on causal strength through weights or time lags. Another advantage is that these approaches can take all relevant variables into consideration and find potentially important causal relationships, which is different from most related studies which only analyze pair-wise causality between two variables. Instead of performing computationally expensive climate model simulations, here we focus solely on an observational-based analysis. Specifically, we examine the sensitivity of causality graphs produced by three methods to different hyperparameters and then compare those graphs with domain knowledge graph.

We found that the outputs of the three algorithms are rather sensitive to the choice of hyperparameters. For example, choosing an only slightly too large regularization parameter can result in NOTEARS or DAG-GNN producing empty graphs, that is not discovering any causal relationships at all. Also the values of the other parameters turned out to be important and outputs for different choices of the hyperparameters can be quite different. Hence, some care must be taken when applying data-driven causality discovery approaches and domain knowledge is indispensable for assessing whether their produced outputs are reasonable.

Compared to domain knowledge graph, the static graphs produced by NOTEARS and DAG-GNN are relatively reasonable. The results from NOTEARS suggest that RH and precipitation dominate sea ice changes among all variables. In the meantime, the sea ice has a large impact on SW and CW. The graph generated by DAG-GNN, however, indicates that the zonal (u10 m) and meridional (v10 m) wind fields are more important for driving sea ice variations than other variables. And there are no atmospheric variables being affected by the sea ice. Note that the edges between u10 m and v10 m, SW and v10 m, CW and v10 m are produced by both NOTEARS and DAG-GNN, which are different from domain knowledge graph, possibly due to the averages over a large domain. As for the temporal graphs, very few edges can be found in TCDF and NOTEARS. In comparison, the DAG-GNN is able to produce more complicated and meaningful results. The sea ice is found to have a delayed impact on itself, but with no causal relationship with any atmospheric processes. This is possibly because sea ice anomaly persistence is much stronger than the connections between sea ice and atmosphere. It is our hope that those causality graphs can be compared with the ones produced by other algorithms as Artificial Intelligence technologies are evolving rapidly. In the meantime, they can be also compared with the causal links captured by physical models for cross-validation.

Based on our analysis, it is still very challenging to directly apply these state-of-the-art data-driven causality discovery approaches to this specific climate topic. However, there are several limitations with current study, which potentially has a large influence on our results. 1) There are large uncertainties in the domain knowledge graph and thus cannot be considered as ground truth. Climate scientists strive to investigate the complex feedbacks between atmosphere and sea ice, but our knowledge in this field is still very limited and controversial. For example, a few recent studies have divergent consensuses on Arctic amplification’s influence on mid-latitude severe winter weather (Blackport et al., 2019; Cohen et al., 2019). 2) We average the atmospheric and sea ice variables within the pan-Arctic domain (north of 60°N) and our analysis is only based on the time-series. However, the causal relationships between atmosphere and sea ice could be regionally dependent. 3) We use the full monthly atmosphere and sea ice records during 1980–2018. The feedbacks between atmosphere and sea ice are highly variable with season, and physical mechanisms work differently with and without sunlight. For example, previous studies pointed out the cloud response to sea ice melt occurs in all seasons except in summer (Kay and Gettelman, 2009; Taylor et al., 2015; Morrison et al., 2018; Huang et al., 2019a). Moreover, the interactions between atmosphere and sea ice may occur at shorter time scales (e.g., daily). 4) The weights among different atmospheric variables are much higher than those between atmosphere and sea ice. Thus, the edges in the latter category could have been filtered out. 6) We only consider the interactions between atmosphere and sea ice in this study. The oceanic processes (e.g., ocean currents, ocean salinity) might also exert large influence on sea ice variations in the Arctic. For example, when salt is ejected into the ocean as sea ice forms, the water’s salinity increases. Therefore, a gradual freshening of the upper Arctic ocean will continue with Arctic sea ice decline (Li and Fedorov, 2021). Our study is a starting point to investigate the atmosphere-sea ice feedback using data-driven causality approaches, and it can be extended to the interactions between atmosphere, ocean, sea ice and even other components (e.g., land ice) in the future. 7) Only one global reanalysis product is used in this study and the results could be more robust if we expand our analysis with different products. It should be noted that the majority of connections considered in this study have been validated by different observational, reanalysis and/or climate models (Ding et al., 2017; Baxter et al., 2019). Thus, we believe that our results may not highly depend on the choice of data sets. Moreover, all results shown in this study are based on detrended and deseasonalized data. We also performed additional experiments with raw data. Based on NOTEARS and DAG-GNN, geopotential heights, clouds, surface longwave, and shortwave flux are found to dominate sea ice changes, which are different from the results shown in Figures 7, 8.

Nevertheless, this is a pioneer study in the application of data-drive causality discovery approaches in the interactions between atmosphere and sea ice. This study will pave the way for us to disentangle the complicated causal relationships in the Earth system, by taking the advantage of cutting-edge data science and Artificial Intelligence technologies. It also provides a good opportunity for climate scientists, data scientists and computer scientists to work together to solve the puzzle in the nature, which will eventually advance our understanding of polar climate system and global climate change.

## Data Availability

The sea ice concentration and extent was obtained from the National Snow and Ice Data Center: http://nsidc.org/data/NSIDC-0051. The ECMWF ERA-5 global reanalysis product can be found via https://cds.climate.copernicus.eu/cdsapp#!/home. The codes and input for three models are available on GitHub: https://github.com/big-data-lab-umbc/cybertraining/tree/master/year-3-projects/team-6.
